# *Schinus terebinthifolius* Essential Oil Attenuates Scopolamine-Induced Memory Deficits via Cholinergic Modulation and Antioxidant Properties in a Zebrafish Model

**DOI:** 10.1155/2019/5256781

**Published:** 2019-12-02

**Authors:** Elena Todirascu-Ciornea, Heba A. S. El-Nashar, Nada M. Mostafa, Omayma A. Eldahshan, Razvan Stefan Boiangiu, Gabriela Dumitru, Lucian Hritcu, Abdel Nasser B. Singab

**Affiliations:** ^1^Department of Biology, Alexandru Ioan Cuza University of Iasi, Iasi 700506, Romania; ^2^Department of Pharmacognosy, Ain Shams University, Cairo 11566, Egypt

## Abstract

*Schinus terebinthifolius* is a plant well recognized for its therapeutic profile such as anti-inflammatory and antitumor activities, promoting antibacterial activity and antioxidant and antidiabetic properties. This study aimed at examining whether *Schinus terebinthifolius* memory-enhancing activities are mediated by cholinergic and brain antioxidant systems in a scopolamine zebrafish model. *Schinus terebinthifolius* essential oil (10, 25, and 50 *μ*L/L) was delivered to zebrafish by immersion in water for 8 days. Memory deficits were induced by scopolamine (100 *μ*M) administration. Zebrafish were divided into seven groups (*n* = 15/group): vehicle group, scopolamine (100 *μ*M) group, *Schinus terebinthifolius* essential oil groups (STF; 10, 25, and 50 *μ*L/L), the imipramine group (IMP; 20 mg/L, as the positive control in the NTT test), and the donepezil group (DP; 10 mg/L, as the positive control in the Y-maze test). Memory status was estimated by the novel tank diving test (NTT) and the Y-maze test and finally was validated by comparison with imipramine (20 mg/L) and donepezil (10 mg/L). Gas chromatography-mass spectrometry (GC-MS) was used to detect oil compounds. Brain levels of acetylcholinesterase (AChE) and antioxidant enzymes were measured. After being exposed to *Schinus terebinthifolius* essential oil, the scopolamine zebrafish exhibited an improvement of memory processes in the NTT and Y-maze tests. The essential oil attenuated the elevated level of AChE and brain oxidative stress. *Schinus terebinthifolius* essential oil was found to support memory formation through the inhibition of the AChE activity and decreasing oxidative stress in the scopolamine-treated zebrafish brains.

## 1. Introduction

Human and animal behavior is regulated by the central cholinergic system [1]. In humans, the memory impairment induced by scopolamine reproduced some aspects of cognitive symptomatology evidenced in Alzheimer's disease (AD) [2], closely related to the degeneration of the cholinergic neurons in the basal forebrain-induced reduction of neurotransmission. Supporting evidence suggested memory deficits induced by scopolamine in zebrafish [3–5]. AChE inhibitors such as donepezil and rivastigmine are involved in the treatment of cognitive symptoms seen in AD [6].

In recent years, the anti-Alzheimer's potential for natural products from medicinal plants has been recognized by the scientific community [7, 8]. Hence, a great deal of research studies focus recently on plants and other natural products used around the world for age-related central nervous system (CNS) diseases [9].

*Schinus L*. (Anacardiaceae) is a genus native to South America particularly to Brazil and comprises about 29 species [10]. Several medicinal properties have been assigned to these plants in addition to their use as ornamental plants [11]. Different parts of *Schinus* plants have been utilized traditionally for the alleviation of several disorders [12]. The essential oils of different *Schinus* species have previously been taken from different locations, and some variations in their chemical components and biological activities have been observed for *Schinus terebinthifolius* [10, 13–15].

Several classes of natural constituents were obtained from this plant, mainly essential oils [16]. Many medicinal properties were reported to the essential oil of *Schinus terebinthifolius*, such as antioxidant, wound healing, antitumor, and antimicrobial activities [17–19]. Moreover, the ethanolic extract of Egyptian *Schinus terebinthifolius* aerial parts exhibited anticholinesterase activity [20]. The pink fruits of *Schinus terebinthifolius* are rich in essential oils (1.5–10%), which was reported to have monoterpenes with high abundance along with some sesquiterpene hydrocarbons [21]. The pharmaceutical industry utilizes the essential oil of *Schinus terebinthifolius* fruits in cosmetics due to its aroma [22], and the Food and Drug Administration (FDA) ranked this essential oil as generally safe (GRAS) [23].

Supporting evidence suggested various biological effects of *Schinus terebinthifolius* Raddi. dos Santos da Rocha et al. [24] reported that *Schinus terebinthifolius* extract exhibited antioxidant and antidiabetic profile in mice mediated by its compounds such as gallic acid, gallotannins, and glycosylated flavonols. Miller et al. [25] demonstrated the vasodilatory activity of *Schinus terebinthifolius* extract in rats and mice and also antioxidant activities, especially in the CNS. The authors attributed these effects mainly to phenolic compounds. Vigo-Pelfrey et al. [26] reported the hepatoprotective effects of *Schinus terebinthifolius* extract against carbon tetrachloride- (CCl_4_-) induced acute hepatotoxicity in rats.

Zebrafish (*Danio rerio*) is a promising *in vivo* tool to model or combat AD [27]. Also, zebrafish exhibited high sensitivity to all major classes of clinically active neurotropic drugs in a similar fashion like humans [28]. Furthermore, complex brain disorders such as depression, autism, psychoses, and cognitive deficits may be successfully analyzed in zebrafish in a cheaper and more powerful manner than in rodents [28]. Supporting evidence suggested that, in zebrafish, scopolamine exhibited memory-reducing effects without causing locomotory deficits and is often used with nootropic and memory-enhancing drugs to study memory formation [29]. Also, recently, our group demonstrated that agathisflavone isolated from *Schinus polygamus* (Cav.) Cabrera leaves improved memory and decreased brain oxidative stress and AChE activity in the scopolamine zebrafish model [5].

The present study is considering to characterize the chemical components of the essential oil isolated from *Schinus terebinthifolius* fruits grown in Egypt and to evaluate the antiamnesic and antioxidant effects of the essential oil against scopolamine using *in vivo* tests and to elucidate the main mechanism of action.

## 2. Materials and Methods

### 2.1. Plant Material

*Schinus terebinthifolius* Raddi fruits were collected during April 2017 from the Garden of Mohamed Ali Palace, Giza, Egypt. Samples were identified by Mrs. Tereize Labib, the Taxonomy Specialist at El-Orman Botanical Garden, Giza, Egypt, and voucher specimen (no. PHG-P-ST-186) has been deposited at Pharmacognosy Department, Faculty of Pharmacy, Ain Shams University.

### 2.2. Extraction of the Essential Oil

The fruits of the plant were immediately hydrodistilled for 4 h using Clevenger-type apparatus. The resulting oil was dried over anhydrous sodium sulfate and stored at 4°C in the dark until use.

### 2.3. Gas Chromatography-Mass Spectrometry (GC-MS)

The GC-MS analyses of the resulting oils were carried out at the Department of Medicinal and Aromatic Plants Research, National Research Center, using TRACE GC Ultra Gas Chromatographs (THERMO Scientific Corp., USA), coupled with a thermomass spectrometer detector (ISQ Single Quadrupole Mass Spectrometer). The GC-MS system was equipped with a TG-5MS column (30 m × 0.25 mm i.d., 0.25 *μ*m film thickness). The analysis was carried out using helium as carrier gas at a flow rate of 1 mL/min and a split ratio of 1 : 10 using the following temperature program: 80°C for 2 min, rising at 5°C/min to 300°C, and is held for 5 min. The injector and detector were held at 280°C. 0.2 *μ*L of diluted samples (1 : 10 hexane, v/v) were always injected. Mass spectra were obtained by electron ionization (EI) at 70 eV, using a spectral range of *m*/*z* 35–500. The components of essential oils were identified by comparison of their Kovats retention indices with those in the literature [30].

### 2.4. Experimental Animals and Maintenance

105 adult male and female, wild-type short-thin zebrafish (*Danio rerio*), 3-4-month-old and 3-4 cm in length, were obtained from an authorized commercial supplier (Pet Product S.R.L., Bucharest, Romania). Animals were randomly divided into groups of 15 fish/10 L tank with a constant 14 : 10 h of the light/dark cycle at 26°C ± 2 and fed twice daily with Norwin Norvitall flake. All fish used in this experiment were observed in quarantine at least one week before use in experimental studies. Acclimated zebrafish were randomly assigned into control, scopolamine (100 *μ*M), and three *Schinus terebinthifolius* essential oil treatment groups (STF; 10, 25, and 50 *μ*L/L). Also, the donepezil group (DP; 10 mg/L) and imipramine (IMP; 20 mg/L) were used as positive controls. The research was accomplished by the Directive 2010/63/EU of the European Parliament and of the Council of 22 September 2010 on the protection of animals. All efforts were made to minimize animal suffering and to reduce the number of animals used.

**Figure 1:**
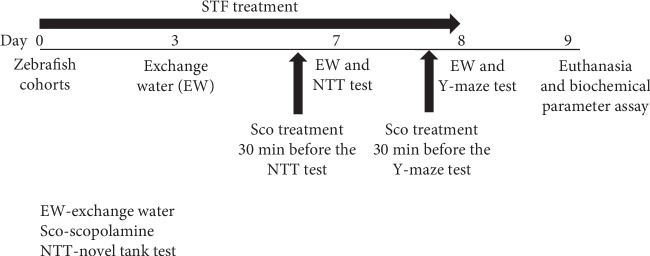
Scheme of the experimental procedure.

The experimental procedure is described in Figure 1. Also, for the behavioral and biochemical parameters assays, we followed the methods of Dumitru et al. [31].

### 2.5. Acute Toxicity Study

An acute toxicity study was determined using 80 fish of both sexes divided into 4 groups as follows: a control group and STF (10, 25, and 50 *μ*L/L) groups. The fish were monitored for two weeks for any symptoms of mortality and toxicity [5].

### 2.6. Novel Tank Diving Test (NTT)

The NTT was used to assess anxiety response in experimental fish according to a method described by Levin et al. [32]. ANY-maze® software (Stoelting Co, USA) and Logitech HD Webcam C922 Prostream camera were used for the automatic tracking of zebrafish behavior. Zebrafish were separately transferred into the tank, and for 6 min, the following variables were recorded: total distance (m), latency to the top (s), time spent in the top (s), time spent in the bottom (s), number of entries in the top, and number of entries in the bottom. In the NTT test, imipramine (IMP; 20 mg/L) was used as the reference drug.

### 2.7. Y-Maze Task

To explore the spatial memory, a Y-maze was used [5]. During 5 min, each fish was singly evaluated [4], and the behavioral endpoints analyzed were as follows: the total distance (m), the percent spontaneous alternation, and the locomotor activity (the number of arm entries). Donepezil (DP; 10 mg/L) was used as the reference drug in the Y-maze test.

### 2.8. Biochemical Parameter Assay

At the end of the experiment, all fish were euthanized by immersion for 10 min in ice water (2–4°C) until the disappearance of opercular movements [33]. The brain samples were dissected out in the ice. Samples were homogenized (1 : 10) in ice-cold 0.1 M potassium phosphate buffer (pH 7.4) and 1.15% KCl using a Potter Homogenizer coupled with Cole-Parmer Servodyne Mixer. The obtained supernatant was used for the biochemical assay, including acetylcholinesterase (AChE) [34], superoxide dismutase (SOD) [35], catalase (CAT) [36], glutathione peroxidase (GPX) [37] specific activities, and malondialdehyde (MDA) level [5]. Protein content was investigated using a bicinchoninic acid (BCA) protein assay kit (Sigma-Aldrich, Germany) using a method as described previously [38].

### 2.9. Statistical Analysis

The results are presented as the mean ± standard error of the mean (SEM) and analyzed using a one-way analysis of variance (ANOVA) followed by Tukey's *post hoc* multiple comparison test, using a *p* < 0.05 level of significance. Pearson correlation coefficient (*r*) was measured to assay the correlation between the behavioral scores, enzymatic activities, and lipid peroxidation. All values were calculated using GraphPad software (GraphPad Prism 7.0, La Jolla, CA, USA).

## 3. Results and Discussion

### 3.1. Acute Toxicity Study

After the administration of STF (10, 25, and 50 *μ*L/L), the zebrafish did not show behavioral changes or other signs of toxicity and mortality, indicating the safety profile of the STF doses. Supporting data provide evidence that the administration of *Schinus terebinthifolius* extract does not induce any toxic effects, which could be an assurance for the medicinal use of this plant in traditional medicine. Lima et al. [39] demonstrated that acute and subacute administration (45 days) of the *Schinus terebinthifolius* bark extract does not produce toxic effects in Wistar rats of both sexes. Affonso et al. [40] reported no signs of toxicity of the essential oil from fruits of *Schinus terebinthifolius* on reproductive functions in male Wistar rats. Our results are following those described by other authors, demonstrating that STF was found to be nontoxic in the tested doses and experimental conditions.

### 3.2. Chemical Composition of the Essential Oil

**Figure 2:**
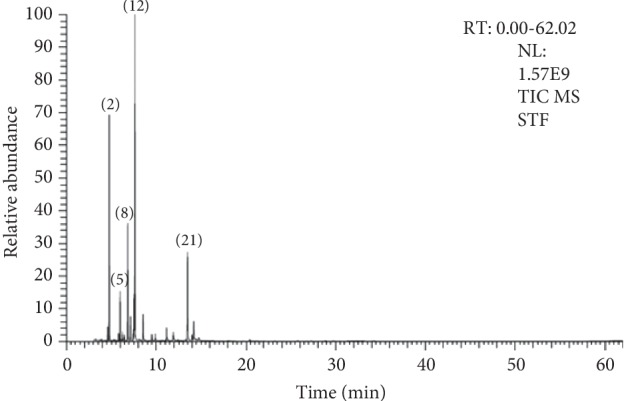
GC-MS chromatogram of essential oil isolated from *Schinus terebinthifolius* fruits grown in Egypt. The major components are highlighted in bold and marked by numbers in Table 1.

A total of 27 chemicals were identified by GC-MS analysis (Figure 2), accounting for 97.22% of the total essential oil (Table 1). The most abundant chemical classes of the oil components were monoterpene hydrocarbons (78.17%), followed by oxygenated monoterpenes (17.54%), sesquiterpene hydrocarbons (0.35%), and oxygenated sesquiterpene (0.33%). The major components of the isolated essential oil were identified as *β*-phellandrene (32.40%), *α*-pinene (16.68%), terpinen-4-ol (11.01%), *α*-phellandrene (10.56%), and *β*-pinene (4.26%), followed by *dl*-limonene (3.74%), *γ*-terpinene (3.03%), *α*-terpineol (2.59%), and *α*-terpinene (2.35%). The chemical composition of our essential oil showed a slight similarity to those reported by other authors. Cole et al. [41] studied the chemical composition of the essential oil from the ripe fruit of *Schinus terebinthifolius* collected on the campus of the Federal University of Espírito Santo (UFES), Goiabeiras, Vitória, Espírito Santo, Brazil. The authors identified 17 compounds accounting for 91.15% of the total oil. Among them, the principal identified compounds were *δ*-3-carene (30.37%), limonene (17.44%), *α*-phellandrene (12.60%), *α*-pinene (12.59%), and *trans*-caryophyllene (1.77%). Maciel et al. [42] identified 37 compounds in the essential oil obtained from *Schinus terebinthifolia* leaves, germacrene B (14.9%), *δ*-cadinene (7.6%), *β*-elemene (7.2%), and *α*-murolol (5.1%) were the most abundant compounds. Ennigrou et al. [43] identified *α*-phellandrene (33.06–36.18%), *α*-pinene (14.85–15.18%), and limonene (6.62–8.79%), being the major components from the essential oil composition of *Schinus terebinthifolius* leaves and twigs collected in the region of Al Ghazala, Ariana, Northern Tunisia. de Lima et al. [44] reported that *Schinus terebinthifolius* contains *α*- and *β*-pinene, Δ^3^-carene, limonene, *α*- and *β*-phellandrene, *p*-cymene, and terpinolene as the main components, together with low amounts of monoterpene alcohols and ketones, sesquiterpene hydrocarbons, alcohols and ketones, and triterpene alcohols and ketones [45]. Another study by Afifi et al. [46] described the *α*-pinene and the *β*-phellandrene as the major components in all the analyzed samples from the *Schinus mole* essential oil growing in Jordan. The observed variation in the chemical composition of the essential oil from *Schinus terebinthifolius* is related to the environmental conditions, the extraction protocols, and the part of the plant submitted to extraction. Based on these results, we found that our essential oil has a chemical composition comparable to those reported by other groups and could support its memory enhancer and antioxidant profile.

The compounds are listed in order of their elution on the DB-5 column. RI, identification based on a comparison of published Kovats retention indices; MS, identification based on mass spectral data.

### 3.3. *Schinus terebinthifolius* Essential Oil Improves Memory Performance

**Figure 3:**
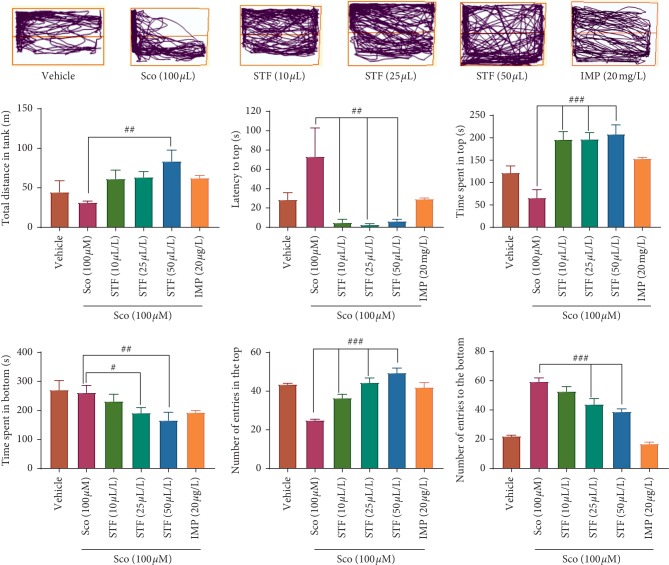
Effects of the *Schinus terebinthifolius* essential oil (STF; 10, 25, and 50 *μ*L/L) and the locomotion tracking pattern and behavioral parameters in different groups in the NTT test. Values are means ± SEM (*n* = 15). For Tukey's *post hoc* analyses, ^##^Sco vs. STF (50 *μ*L/L): *p* < 0.01 for total distance in tank, ^##^Sco vs. STF (10 *μ*L/L): *p* < 0.001, ^##^Sco vs. STF (25 *μ*L/L): *p* < 0.001, and ^##^Sco vs. STF (50 *μ*L/L): *p* < 0.001 for latency to top, ^###^Sco vs. STF (10 *μ*L/L): *p* < 0.0001, ^###^Sco vs. STF (25 *μ*L/L): *p* < 0.0001, and ^###^Sco vs. STF (50 *μ*L/L): *p* < 0.0001 for time spent in top, ^#^Sco vs. STF (25 *μ*L/L): *p* < 0.01 and ^##^Sco vs. STF (50 *μ*L/L): *p* < 0.001 for time spent in bottom, ^###^Sco vs. STF (10 *μ*L/L): *p* < 0.0001, ^###^Sco vs. STF (25 *μ*L/L): *p* < 0.0001, and ^###^Sco vs. STF (50 *μ*L/L): *p* < 0.0001 for number of entries in the top and ^###^Sco vs. STF (25 *μ*L/L): *p* < 0.0001 and ^###^Sco vs. STF (50 *μ*L/L): *p* < 0.0001 for number of entries to the bottom.

One-way ANOVA demonstrated overall powerful changes of the total distance (*F* (5, 84) = 3.23, *p* < 0.01), on the latency to top (*F* (5, 84) = 4.56, *p* < 0.001), on the time spent in top (*F* (5, 84) = 12.11, *p* < 0.0001), on the time spent in bottom (*F* (5, 84) = 2.64, *p* < 0.01), on the number of entries in the top (*F* (5, 84) = 20.35, *p* < 0.0001), and on the number of entries to the bottom (*F* (5, 84) = 44.98, *p* < 0.0001). In addition, zebrafish exposed to scopolamine (100 *μ*M) displayed anxiogenic-like profile, as evidenced by a significant increase of the latency to top (*p* < 0.001) and of the number of entries to the bottom (*p* < 0.0001) and a significant decrease of the time spent in top (*p* < 0.001) and of the number of entries to the top (*p* < 0.001) as compared to the vehicle group (Figure 3). Furthermore, STF attenuates the effects induced by scopolamine in a dose-dependent manner as compared to imipramine effects, collectively suggesting anxiolytic effects. Accordingly, locomotion tracking plots in zebrafish exposed to scopolamine were altered, whereas STF restored locomotion patterns nearly like an imipramine-treated group.

**Figure 4:**
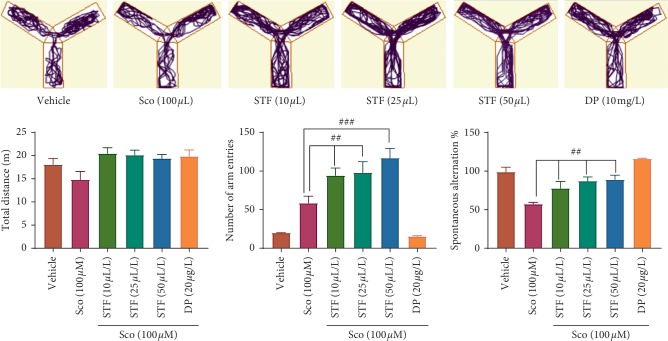
Effects of the *Schinus terebinthifolius* essential oil (STF; 10, 25, and 50 *μ*L/L) on the locomotion tracking pattern and behavioral parameters in different groups in the Y-maze test. Values are means ± SEM. (*n* = 15). For Tukey's post hoc analyses, ^##^Sco vs. STF (10 *μ*L/L): *p* < 0.001, ^##^Sco vs. STF (25 *μ*L/L): *p* < 0.001, and ^###^Sco vs. STF (50 *μ*L/L): *p* < 0.0001 for number of arm entries, ^##^Sco vs. STF (10 *μ*L/L): *p* < 0.001, ^##^Sco vs. STF (25 *μ*L/L): *p* < 0.001, and ^##^Sco vs. STF (50 *μ*L/L): *p* < 0.001 for spontaneous alternation %.

Furthermore, to assess the effects of STF on memory, the Y-maze test was employed (Figure 4). One-way ANOVA revealed representative changes of the total distance (*F* (5, 84) = 2.66, *p* < 0.01), on the number of arm entries (*F* (5, 84) = 20.93, *p* < 0.001), and on the percentage of the spontaneous alternation (*F* (5, 84) = 13.45, *p* < 0.0001). Also, the scopolamine treatment-induced memory deficits as evidenced by a significant decrease in the spontaneous alternation percentage (*p* < 0.0001) as compared to the vehicle group. Also, the treatment with scopolamine promoted decreased locomotion tracking plots that were prevented by STF treatment nearly to the donepezil group. Our results suggested that *Schinus terebinthifolius* essential oil possesses anxiolytic and memory supporting profile, which is in agreement with the literature where *Schinus terebinthifolius* administration exhibited the neuroprotective effects possibly mediated by its antioxidant activity in a rotenone rat model of Parkinson's disease [7]. In addition, the improvement of memory deficits as a result of STF administration could be attributed to not only the presence in high amounts of the active compounds such as *β*-phellandrene, *α*-pinene, terpinen-4-ol, and *α*-phellandrene but also to the presence of other components in small amounts but with high activity such as *β*-pinene, followed by dl-limonene, *γ*-terpinene, *α*-terpineol, and *α*-terpinene or synergy between them. Lee at al. [47] demonstrated that *α*-pinene exhibited neuroprotective effects against memory impairment induced by scopolamine in C57BL/6 mice by increasing the mRNA expression of choline acetyltransferase in the cortex and the activity of the antioxidant enzymes in the hippocampus via activation of NF-E2-related factor 2. *α*-Phellandrene, a cyclic monoterpene presented in our STF, was reported to attenuate the inflammatory response through neutrophil migration modulation and mast cell stabilization in male Wistar rats or Swiss mice [48]. Machado et al. [49] identified the presence of high content of triterpenes in the chemical composition of the *Schinus molle* leaves extract. Also, the authors evidenced the antidepressant-like effect of the *Schinus molle* leaves extract in mice being dependent by its interaction with the serotonergic (5-HT_1A_, 5-HT_2A_, and 5-HT_3_ receptors), noradrenergic (*α*_1_ and *α*_2_-receptors), and dopaminergic (*D*_1_ and *D*_2_ receptors) systems. Piccinelli et al. [50] demonstrated antidepressive actions of the essential oil from *Schinus terebinthifolius* fruits in rats mediated by (R)-(+)-limonene and *α*-phellandrene. However, recently, we demonstrated that *Schinus polygamus* (Cav.) Cabrera leaves extract reduced cognitive deficits in scopolamine-treated zebrafish, mainly due the presence of the agathisflavone, a natural biflavonoid [5]. Our research provides rational use of the STF in the direction of memory deficits, anxiety, depression, and connected dementia.

### 3.4. *Schinus terebinthifolius* Essential Oil Exhibited Antiacetylcholinesterase Activity

**Figure 5:**
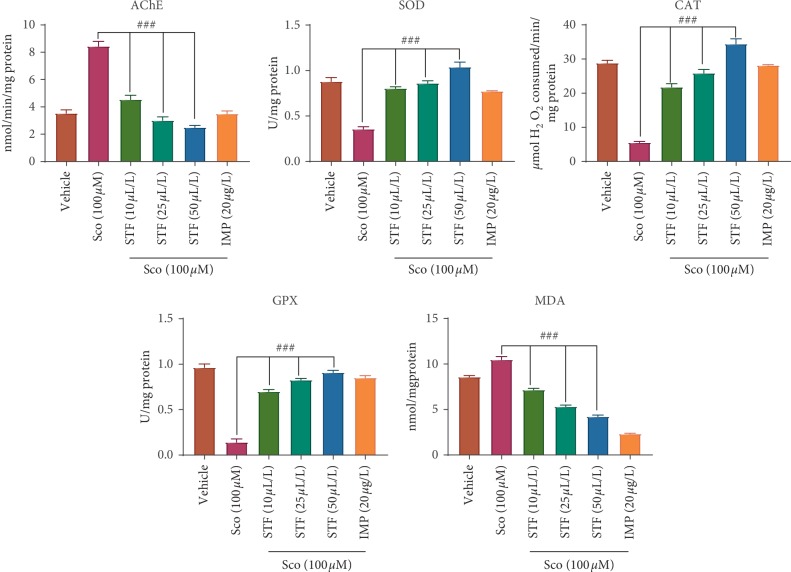
Effects of the *Schinus terebinthifolius* essential oil (STF; 10, 25, and 50 *μ*L/L) on AChE, SOD, CAT, and GPX specific activities and MDA level in different groups. Values are means ± SEM. (*n* = 15). For Tukey's post hoc analyses, ^###^Sco vs. STF (10 *μ*L/L): *p* < 0.0001, ^###^Sco vs. STF (25 *μ*L/L): *p* < 0.0001, and ^###^Sco vs. STF (50 *μ*L/L): *p* < 0.0001 for AChE, ^###^Sco vs. STF (10 *μ*L/L): *p* < 0.0001, ^###^Sco vs. STF (25 *μ*L/L): *p* < 0.0001, and ^###^Sco vs. STF (50 *μ*L/L): *p* < 0.0001 for SOD, ^###^Sco vs. STF (10 *μ*L/L): *p* < 0.0001, ^###^Sco vs. STF (25 *μ*L/L): *p* < 0.0001, and ^###^Sco vs. STF (50 *μ*L/L): *p* < 0.0001 for CAT, ^###^Sco vs. STF (10 *μ*L/L): *p* < 0.0001, ^###^Sco vs. STF (25 *μ*L/L): *p* < 0.0001, and ^###^Sco vs. STF (50 *μ*L/L): *p* < 0.0001 for GPX and ^###^Sco vs. STF (10 *μ*L/L): *p* < 0.0001, ^###^Sco vs. STF (25 *μ*L/L): *p* < 0.0001, and ^###^Sco vs. STF (50 *μ*L/L): *p* < 0.0001 for MDA.

To further elucidate the potential mechanism of the STF against scopolamine-induced memory deficits, the brain AChE activity was firstly evaluated. One-way ANOVA demonstrated overall significant effects on AChE specific activity (*F* (5, 84) = 68.57, *p* < 0.0001) (Figure 5). Treatment with scopolamine generates a significant increase in AChE activity in the brain as compared to the vehicle group (*p* < 0.0001). In contrast, the administration of the STF significantly reversed the effects of scopolamine on AChE activity (*p* < 0.0001). The effects of STF were consistent with those of donepezil. Donepezil significantly improved memory in the Y-maze test and showed inhibition of AChE. These results suggest that STF could protect against scopolamine-induced dysfunction of the cholinergic system.

AChE overexpression was evidenced as an important indicator of the cholinergic system dysfunction that could play a pivotal role in AD pathogenesis [51]. However, AD therapy implies AChE inhibitors and thereby enhances cholinergic neurotransmission [52]. Murray et al. [53] reported that the essential oils from *Schinus areira* L. and *Schinus longifolia* (Lindl.) Speg. showed AChE inhibition. Among them, *Schinus longifolia* essential oil exhibited high AChE inhibition with an IC_50_ value of 20 ± 1 *μ*g/mL. The STF used in the present study contains high levels of monoterpenes, like *α*-pinene (16.68%), *α*-phellandrene (10.56%), and *β*-phellandrene (32.40%) that could explain the observed AChE inhibitory activity and antioxidant activity, respectively.

### 3.5. *Schinus terebinthifolius* Essential Oil Restored the Brain Antioxidant Status

To support the memory enhancement and anti-AChE activities of STF in the scopolamine-treated rats, the brain antioxidant status was assessed secondly. Antioxidant enzymes including SOD, CAT, and GPX play a decisive role in the reduction of oxidative stress [54].

One-way ANOVA demonstrated overall significant effects on SOD (*F* (5, 84) = 50.33, *p* < 0.0001), CAT (*F* (5, 84) = 60.20, *p* < 0.0001), and GPX (*F* (5, 84) = 59.50, *p* < 0.0001) specific activities (Figure 5).

SOD, CAT, and GPX enzyme activity levels dramatically decreased as compared to the vehicle group after exposure to scopolamine (*p* < 0.0001), whereas the administration of the STF could restore the antioxidant status in the scopolamine-treated groups in a dose-dependent manner (*p* < 0.0001). Also, one-way ANOVA revealed significant effects on the MDA level (*F* (5, 84) = 24.28, *p* < 0.0001). At the same time, the level of MDA increased in the scopolamine-treated groups (*p* < 0.0001) as compared to the vehicle group, while exposure to STF dramatically decreased the level of MDA in a dose-dependent manner in the scopolamine-treated groups (*p* < 0.0001).

It has been reported that the brain of the patients suffering from AD presents a significant extent of oxidative damage [30]. Moreover, oxidative stress can induce cellular apoptosis in brain and cognitive impairment [55]. The activation of the antioxidant enzymes such as SOD and GPX could scavenge reactive oxygen species and protect cells against oxidative damage [56]. In our study, scopolamine decreased the antioxidant enzyme activity levels such as SOD, CAT, and GPX and increased the MDA level in the zebrafish brain. Thus, scopolamine created an imbalance between antioxidant and oxidant defense systems which may be responsible for observed impairment of memory in zebrafish. However, the essential oil restored the antioxidant status as evidenced by increased SOD, CAT, and GPX activity in scopolamine-treated zebrafish that parallels with a decrease of MDA level in the zebrafish brain. The results indicated that STF exhibits neuroprotective effects against oxidative stress, which is correlated with the previous studies. Silva et al. [57] demonstrated *in vitro* antioxidant, antiproliferative, and *in vivo* anti-inflammatory activities of *Schinus terebinthifolius* due to the presence of phenolic constituents. However, Dumitru et al. [31] already reported that *Schinus polygamus* (Cav.) Cabrera leaves extract is capable to control brain oxidative status in a scopolamine zebrafish model through agathisflavone, a natural biflavonoid that could restore the antioxidant enzyme activities. Our results agree with the behavioral results. Thus, we presumed that the modification of the cholinergic neuronal system and the reduction of oxidative stress is the underlying mechanism to the cognitive enhancement effect of STF in scopolamine-treated rats.

Using the Pearson correlation coefficient, which quantifies the linear association between two quantitative variables, it was possible to estimate the relationships between different behavioral scores and biochemical parameters [58]. Our results revealed strong correlations between behavioral scores, enzymatic activities, and lipid peroxidation. Moreover, when the Pearson correlation coefficient (*r*) was evaluated, significant correlations between the spontaneous alternation percentage vs. MDA, the time spent in top vs. MDA, time spent in bottom vs. MDA, and the number of entries to the top vs. MDA, AChE vs. MDA, SOD vs. MDA, CAT vs. MDA, and GPX vs. MDA were reported (Table 2).

The negative correlation coefficient indicates that the lower is the value of MDA and the higher are the values of behavioral scores and antioxidant enzymes.

A significant correlation between potential antioxidant and anxiolytic effects of (+)-limonene epoxide has been evidenced in mice [59]. Alzate et al. [60] evidenced a correlation between the free radical-scavenging activity exhibited by *Bomarea* species and the phenolic content in the extracts.

The correlation coefficients that were assessed in the current study indicate that the antioxidant profile possessed by STF as a result of high amounts of monoterpenes is well correlated with the improvement of memory in behavioral tests (NTT and Y-maze) along with the decrease of AChE activity. Thus, STF exhibits neuroprotection against scopolamine-induced oxidative stress generation in the zebrafish brain.

## 4. Conclusions

In conclusion, our results contribute to clarify the pharmacology of *Schinus terebinthifolius* essential oil in the zebrafish model of scopolamine, by demonstrating its memory-enhancing effects via cholinergic modulation and antioxidant properties. Further studies are required for a possible application of *Schinus terebinthifolius* essential oil to improve cognition.

## Figures and Tables

**Table 1:** The chemical composition of essential oil isolated from the fruits of *Schinus terebinthifolius*.

No.	Compound	Molecular formula	Retention index		Relative composition (%)
			Calculated	Reported	
1.	Thujene	C_10_H_16_	923	930	1.08
2.	***α*-Pinene**	C_10_H_16_	931	939	**16.68**
3.	Camphene	C_10_H_16_	950	954	0.17
4.	Sabinene	C_10_H_16_	972	975	0.68
5.	***β*-Pinene**	C_10_H_16_	977	979	**4.26**
6.	*α*-Myrcene	C_10_H_16_	987	990	0.92
7.	Decane	C_10_H_22_	994	1000	0.56
8.	***α-*Phellandrene**	C_10_H_16_	1005	1002	**10.56**
9.	*α*-Terpinene	C_10_H_16_	1017	1017	2.35
10.	*o*-Cymene	C_10_H_14_	1027	1026	0.74
11.	*dl*-Limonene	C_10_H_16_	1029	1029	3.74
12.	***β*-Phellandrene**	C_10_H_16_	1030	1029	**32.40**
13.	*α*-Ocimene	C_10_H_16_	1045	1039	0.25
14.	*γ*-Terpinene	C_10_H_16_	1057	1059	3.03
15.	*cis*-Sabinene hydrate	C_10_H_18_O	1072	1070	0.09
16.	*α*-Terpinolene	C_10_H_16_	1083	1088	0.75
17.	Undecane	C_11_H_24_	1093	1100	0.85
18.	*cis*-*p*-Menth-2-en-1-ol	C_10_H_18_O	1122	1121	1.66
19.	*Trans-*1-Methyl-4-(1-methylethyl)-2-cyclohexen-1-ol	C_10_H_18_O	1142	1139	1.24
20.	Isoborneol	C_10_H_18_O	1156	1160	0.11
21.	**Terpinen-4-ol**	C_10_H_18_O	1178	1177	**11.10**
22.	*α*-Terpineol	C_10_H_18_O	1193	1188	2.59
23.	*trans*-Piperitol	C_10_H_18_O	1206	1208	0.48
24.	*α*-Terpinyl acetate	C_12_H_20_O_2_	1345	1349	0.27
25.	Germacrene D	C_15_H_24_	1482	1485	0.11
26.	Germacrene B	C_15_H_24_	1553	1561	0.24
27.	10-Epi-*γ*-eudesmol	C_15_H_26_O	1619	1623	0.31
Monoterpene hydrocarbons					**78.17**
Oxygenated monoterpenes					**17.54**
Sesquiterpene hydrocarbons					**0.35**
Oxygenated sesquiterpenes					**0.31**
Others					**0.85**
Total identified					**97.22**

**Table 2:** Correlation data between the different parameters measured in the study (behavioral and biochemical parameters).

	Biochemical parameters	Pearson correlation coefficient (*r*)	Significance level (*p*)
Behavioral scores			
Spontaneous alternation (%)	MDA	−0.563	*p* < 0.01
Time spent in top (s)	MDA	−0.653	*p* < 0.001
Time spent in bottom (s)	MDA	0.541	*p* < 0.01
Number of entries to the top	MDA	−0.512	*p* < 0.01
Biochemical parameters			
AChE	MDA	0.724	*p* < 0.001
SOD	MDA	−0.586	*p* < 0.01
CAT	MDA	−0.713	*p* < 0.001
GPX	MDA	−0.630	*p* < 0.001

## Data Availability

The data used to support the findings of this study are available from the corresponding author upon request.
